# ASO Author Reflections: Does Perioperative Radiotherapy have the Potential to Improve Disease-Specific Survival in Patients with Retroperitoneal Liposarcoma?

**DOI:** 10.1245/s10434-024-16830-4

**Published:** 2025-01-04

**Authors:** Alexander Wilhelm, Benjamin Wiesler, Beat P. Müller

**Affiliations:** 1 Department of Surgery, Clarunis – St. Clara Hospital & University Hospital Basel, Basel, Switzerland; 2 Surgical Outcome Research Center Basel, University Hospital Basel, University Basel, Basel, Switzerland

## Past

The potential benefits of neoadjuvant radiotherapy in patients with localized retroperitoneal sarcoma who undergo surgery have been called into question by the randomized STRASS trial.^1^ However, a subgroup analysis of patients with retroperitoneal liposarcoma suggested a possible beneficial effect of neoadjuvant radiotherapy on local recurrence rate, which was supported by a pooled analysis of STRASS trial and off-trial (STREXIT) data.^2^ The purpose of this study was to evaluate whether this beneficial effect of perioperative radiotherapy in liposarcoma on local recurrence rates translates into an improvement in disease-specific survival.

## Present

We conducted a population-based study comprising patients who underwent surgery for localized retroperitoneal liposarcoma between 2004 and 2020. The SEER-17 database, which encompasses 26.5% of the U.S. population, was used for this analysis. A total of 1692 patients were included of whom 393 had undergone perioperative radiotherapy. Following propensity score matching, COX proportional hazard regression analysis and Kaplan-Meier survival analysis demonstrated that perioperative radiotherapy did not result in improved disease-specific survival in patients with retroperitoneal liposarcoma.^3^ In multivariable-adjusted COX regression, the strongest predictors of impaired disease-specific survival were patient age of 80 years or greater compared with less than 60 years, tumor size > 10 cm or unknown size compared with < 10 cm, and tumor grading of G3 in comparison to G1/G2.

## Future

Retroperitoneal liposarcoma is a rare disease, and some patients may be long-time survivors, so conducting a prospective randomized trial to assess disease-specific survival or overall survival following perioperative radiotherapy in these patients is extremely difficult. One possibility to evaluate disease-specific survival and to further confirm findings from prospective (randomized) trials is using existing population-based real-world databases, e.g., the SEER database. Some centers routinely perform neoadjuvant radiotherapy in patients with G1/2 retroperitoneal liposarcoma following the results of the STRASS and STREXIT trial, other centers more selectively chose to perform radiotherapy. In light of the current analysis, we suggest that the routine use of perioperative radiotherapy in patients with localized and resectable retroperitoneal liposarcoma may be questioned. However, the results of our study should be interpreted with caution due to the limitations of the SEER database.
